# A Case of Colic Attended with Obstinate Constipation; Stercoraceous Vomiting; Recovery

**Published:** 1849-01

**Authors:** John P. Dromgoole

**Affiliations:** Rutherford County, Tennessee; Millersburg


ART. II.—*A Case of Colic attended with obstinate Constipation; Stercoraceous Vomiting; Recovery.* By JOHN P. DROMGOOLE, M. D., of Rutherford County, Tennessee.

On Sunday the 15th of October last, I was called to see Mr. T. C., whom I found laboring under, what I thought a severe attack of cramp colic, which came on soon after supper the evening before. His pulse was 90 and full; tongue coated with fur; complexion bilious; complaining of very severe pain in the region of the colon; bowels costive; surface of body cold and clammy; no swelling in the region of the pain; no tenderness upon pressure. I gave him 25 grs. calomel, combined with 15 grs. jalap, hoping it would produce an evacuation from his bowels, and relieve them of their irritating contents.

About 12 o'clock that night I was again called to him in great haste, and found all the symptoms much aggravated, his medicine not having operated. I gave him 15 grs. of jalap, and 20 grs. calomel followed by senna tea, which proved equally ineffectual. The pain being so excrutiating I gave him morphia, and used at the same time hot fomentations, hot salt, hot ashes, friction, &c., over the seat of pain, but all without any material abatement of the symptoms.

16th. Patient no better; symptoms even more alarming; vomits up large quantities of bile; medicine has produced no effect. I gave him large quantities of ext. colocynth in the evening followed by castor oil, senna, manna, gamboge, &c., without producing any effect upon his bowels. Just before night, ordered several simple injections but with no better results. Same night, ordered injections, of ext. colocynth, manna, olive oil, spts. turpentine, &c., but to no advantage.

17th. Patient's stomach very irritable; vomits something which very much resembles coffee grounds. Warm bath used, but with no relief to the symptoms. Blister to the epigastrium, and venesection, under which his pulse became frequent and feeble. Towards midnight he became almost pulseless, his eyes sunken; surface bedewed with a cold clammy perspiration; very great pain in abdomen; convulsive movements of the limbs and body. Under stimulants he rallied a little before morning.

18th. Medicine has produced no effect on his bowels. The only medicine now used is morphia and opium. In the evening he commenced vomiting large quantities of stercoraceous matter, which increased till about 11 o'cock at night, at which time, while vomiting, he had an evacuation from the bowels.

19th. Patient much better; pain greatly alleviated, yet stomach very irritable; vomits every thing taken into the stomach, except small doses of sodaic powders.

20th. Complains greatly of his stomach; very much pain, pulse 100, full and strong; tenderness in the epigastrium. I applied a large blister over the region of the stomach, gave mucilaginous drinks, sodaic powders, &c.

22d. Patient much better; slept a little; stomach not so irritable; pulse 80 and not so full.

23d. To-day his abdomen became very tender upon pressure, and somewhat swollen; very offensive dejections from bowels. Applied a blister over the whole abdominal surface; mucilaginous drinks; sodaic powders.

24th. Patient is much relieved.

25th. Urgent diarrhœa commenced, and threatened to carry the patient off. Dejections watery and offensive; tongue foul; pulse 110 and weak. Administered calomel, chalk, acetate of lead, opium, kino, galls, &c.

26th. Diarrhœa unabated, and stools have become bloody; bleeding also from the gums and nose; bloody urine; involuntary discharges; extensive exhaustion. Ordered 1 gr. nitrate of silver every hour for six hours.

27th. Patient feels much better though very weak, and almost pulseless; diarrhœa arrested; tongue becoming clean, some pain.

28th. Typhoid symptoms came on. Active and immediate stimulation resorted to and persevered in till the 30th, at which time he was a little better.

30th. Pulse 90, weak and regular, tongue a little coated and red on edges; some appetite, complains greatly of his back; abdomen sore; bowels too loose, but checked by the chalk mixture.

Patient has been gradually improving ever since, and is now walking about, though still feeble.

The above is certainly a remarkable case. The first question that naturally arises, is as to its pathology. What was the nature of the obstruction? Was it spasm, a stricture, a hernia, some foreign body in the bowel, or was it intussusception? I confess, a satisfactory answer has not suggested itself to my mind. Recovery has seldom taken place under a more alarming combination of symptoms—stercoraceous vomiting, convulsions, involuntary evacuation of bowels and bladder, bloody urine, and hemorrhage from the nose and gums. It may admit of doubt whether the active medication in the first stage of the disease was not prejudicial, and had not some agency in the development of the train of symptoms that succeeded. I should resort earlier to the lancet in a similar case, and, from the increasing testimony in its favor, should certainly make trial of chloroform as an anodyne, instead of relying upon morphia and opium. The doses in which I administered the nitrate of silver were large, but the result seemed to justify the practice; for the diarrhœa, which had proved intractable under all the ordinary astringents, yielded promptly to this remedy.

Millersburg, Nov. 27th, 1848.

